# Analysis of Language Translations of State Governments' Coronavirus Disease 2019 Vaccine Websites

**DOI:** 10.1089/heq.2021.0189

**Published:** 2022-09-22

**Authors:** Nicole C. Tensmeyer, Nathan N.L. Dinh, Landy T. Sun, Corey B. Meyer

**Affiliations:** Gryphon Scientific, Takoma Park, Maryland, USA.

**Keywords:** COVID-19 vaccine, vaccination campaign, language translation, language access, limited English proficiency

## Abstract

**Introduction:**

During the coronavirus disease 2019 (COVID-19) vaccination campaign, non-English-communicating individuals have faced inequities in access to resources for vaccine education and uptake. We characterized the language translation status of states' COVID-19 vaccine websites to inform discussion on the sufficiency of translated information and strategies for expanding the availability of multilingual vaccine information.

**Methods:**

We identified the primary COVID-19 vaccine website for all 50 states, the District of Columbia, and the federal government (“jurisdictions”) and determined the languages into which information about obtaining the vaccine (access) and vaccine safety and efficacy had been translated, as of October 2021. We compared these findings with data from the American Community Survey to determine how many individuals had these online resources available in their primary language.

**Results:**

Only 56% of jurisdictions provided professionally translated information about COVID-19 vaccine safety and efficacy, and only 50% provided professionally translated information about how to register for or obtain the COVID-19 vaccine, in at least one language. Consequently, ∼26 million Americans may not have accurate vaccine safety and efficacy information available, and ∼29 million Americans may not have vaccine access information available, from their jurisdiction in their primary language. Furthermore, translated information often was limited in scope and/or number of languages provided.

**Conclusion:**

Translation of COVID-19 vaccine information on state government websites currently is insufficient to meet the needs of non-English-communicating populations. This analysis can inform discussions about resource needs and operational considerations for adequate provision of multilingual, critical health information.

## Introduction

Research throughout the coronavirus disease 2019 (COVID-19) pandemic has shown that non-English-communicating individuals in the United States are more likely to become infected with and die from COVID-19 than individuals proficient in English.^1–3^ Since COVID-19 vaccines are a critical tool for protecting vulnerable populations, ensuring that the 25 million Americans with limited English proficiency (LEP) have knowledge about and access to COVID-19 vaccines is an important facet of addressing health inequity issues faced by this population.^4^

State and local public health departments (PHDs) have taken a leading role in coordinating COVID-19 vaccine distribution and promoting uptake. As part of their vaccination campaigns, PHDs created websites featuring the latest information about COVID-19 vaccines, which help to combat misinformation regarding vaccine safety and efficacy. Within these websites, states also provide information about how to obtain the vaccine in their jurisdictions to facilitate access.

Many studies have demonstrated that linguistic barriers contribute to health inequities experienced by non-English-communicating populations.^5–8^ Federal language access laws, such as Title VI of the Civil Rights Act of 1964 and Executive Order 13166, require anyone receiving federal funding to provide meaningful access to federal resources to individuals who do not speak English. This legislation provides guidelines for the types of information that must be translated but does not define specific population thresholds for determining which languages must be provided. Some states and localities also have language access policies that reinforce and support implementation of the federal requirements.^9–11^ Many state/local policies use a 5% population threshold to determine whether to provide language services, although some use a numeric threshold (i.e., number of language speakers).^10,12–14^

Research examining how translation of government websites influences health disparities is lacking. However, health equity experts widely recommend the provision of multilingual health information resources on government websites as part of a comprehensive strategy for outreach to non-English-speaking communities about health issues.^5,6,15^

Despite legal requirements, translation of government COVID-19 vaccine websites has been limited and heavily reliant on automated translation programs such as Google Translate (GT).^16–19^ Numerous studies have documented errors in machine translation of public health and medical information that reduce accuracy, understandability, and usability of the information.^20–22^ Consistent with this research, multiple lay press articles have documented serious errors in machine-translated COVID-19 vaccine information on PHD websites.^17,23,24^ Anecdotal evidence suggests that the lack of linguistically and culturally appropriate information about COVID-19 vaccines hampers access for many non-English-communicating Americans, which likely exacerbates health disparities they experience.^18,19,25–28^

A better understanding of the language coverage of government COVID-19 websites is needed to inform efforts to improve COVID-19 vaccine uptake among non-English-communicating populations and improve language accessibility during future public health emergencies. Therefore, we conducted a comprehensive analysis of state COVID-19 websites for availability of professionally translated information about the COVID-19 vaccine and how to obtain it. We then evaluated how state-provided translations compared with languages spoken by their respective populations based on American Community Survey (ACS) data on language use. Our analysis showed that professional translations of states' COVID-19 vaccine websites were insufficient, leaving as many as 29 million Americans without these resources available in their primary language. To inform discussion on resources needed for translation, we estimated how many and which language translations would be required in each state to provide for the majority of their non-English speakers.

## Methods

### COVID-19 website identification and evaluation

We identified the primary COVID-19 vaccine websites for all 50 states, the District of Columbia (D.C.), and the Federal government (collectively referred to as “jurisdictions”). Websites were identified by searching each jurisdiction's PHD website and performing an independent search using the terms “[JURISDICTION]” and “COVID-19 vaccine.” Each website was thoroughly explored to identify and evaluate translated information in two categories: (a) general information about the safety and efficacy of the COVID-19 vaccine (called *informational* resources), and (b) information about how to obtain the vaccine, including a registration webpage (state-run or the Center for Disease Control and Prevention's Vaccine Administration Management System) and/or information about community sites/clinics and/or third parties providing the vaccine (called *access* resources).

Information published directly on websites and in documents that could be accessed directly was evaluated. We collected data on whether translated informational and access resources were provided; the languages provided; and whether the translations were professional or machine generated.

Resources that were machine translated but professionally reviewed were counted as professional translations, as studies have shown that postediting of automated translations increases accuracy and quality.^29–31^ PHDs were contacted to verify if their resources were professionally translated or reviewed. For the nine jurisdictions that did not respond, we analyzed several facets of each translated webpage to determine whether its translations were human or machine generated: changes in the text of embedded images, source code, including non-English text, changes in punctuation, and changes in page formats between English and non-English pages. To get “credit” for a translation, a translated resource needed to include basic information about vaccine safety and efficacy (informational resources) or options for obtaining the vaccine (access resources) but did not need to be identical to the corresponding English-language resource. All data were collected from October 12 to 19, 2021.

All websites were independently identified and evaluated by two reviewers to ensure the evaluation was comprehensive and consistent. Supplementary Table S1 provides a list of websites evaluated for each jurisdiction and additional notes on evaluation metrics.

### Analyses of translation sufficiency

To assess the language coverage of jurisdictions' multilingual COVID-19 vaccine resources, we used the 2019 ACS “Language other than English spoken at home” dataset, which tabulates the estimated number of speakers for 139 languages in each of the 50 states and D.C. We used ACs' definitions of languages in this study, although those designated as “Other languages” were not counted as individual languages because many of these include multiple distinct languages.

We analyzed which languages would be expected if each jurisdiction were to provide translations for every language with primary speakers making up at least 5%, 0.5%, or 0.07% of the jurisdiction's population. Five percent is the most common threshold used in states' language access policies to determine whether a state agency is required to provide language services.^10,11,13,14^ The 0.07% threshold was derived from D.C.'s and Hawaii's language accessibility laws, which require translations for languages with 500 and 1000 speakers, respectively, representing 0.07% of their respective populations.^12,32^ We also applied an intermediary 0.5% threshold as we recognized that the 0.07% threshold may be impractical for states to meet, while the 5% threshold leaves many non-English speakers without translated resources. We then estimated the number of individuals whose primary language is not English for whom professional translations of COVID-19 resources are available in their primary language from their jurisdiction's website.

Data were analyzed in Microsoft Excel and data visualization was performed using Tableau Software.

This research was exempt from IRB review by the IRB administrator at Gryphon Scientific, LLC.

## Results

### Translation status of COVID-19 websites

Of the 52 jurisdictions, 29 (56%) provided professional translations of informational resources regarding the safety and efficacy of the COVID-19 vaccine in at least one language. Of these, 13 provided machine-generated translation(s) for additional languages, and an additional 19 jurisdictions integrated GT widgets into their vaccine information webpages or provided machine-generated translations of that information. Translations of vaccine access resources were more limited, with 27 (52%) jurisdictions providing professional translations in at least one language, 13 of which also provided machine-generated translations. An additional 17 jurisdictions provided only machine-generated translations of access resources. Only 22 (42%) jurisdictions provided professional translations for both informational and access resources. These data are summarized in Table 1.

**Table 1.** Summary of Resources Provided by Each Jurisdiction

Jurisdiction	Informational resources	Access resources
Alabama	None	None
Alaska	None	None
Arizona	Original	Original
Arkansas	Machine-Generated and Original	Machine-Generated and Original
California	Machine-Generated and Original	Original
Colorado	Original	Original
Connecticut	Machine-Generated	Machine-Generated and Original^a^
Delaware	Original	None
District of Columbia	Machine-Generated	Machine-Generated
Federal	Original	Original
Florida	Original	Original
Georgia	Machine-Generated and Original	Machine-Generated and Original
Hawaii	Machine-Generated	Machine-Generated and Original^a^
Idaho	Machine-Generated	Machine-Generated
Illinois	Machine-Generated	Machine Generated and Original
Indiana	Machine-Generated	Machine-Generated and Original
Iowa	Machine-Generated	Machine-Generated
Kansas	Machine-Generated	Machine-Generated and Original
Kentucky	Machine-Generated	Machine-Generated
Louisiana	Machine-Generated and Original	Machine-Generated and Original
Maine	Machine-Generated	Machine-Generated
Maryland	Machine-Generated	None
Massachusetts	Machine-Generated and Original	Machine-Generated
Michigan	Original	None
Minnesota	Original	Original^b^
Mississippi	Machine-Generated	Machine-Generated
Missouri	Machine-Generated	Machine-Generated
Montana	Machine-Generated	Machine-Generated
Nebraska	Machine-Generated and Original	Machine-Generated and Original
Nevada	Machine-Generated	Machine-Generated
New Hampshire	Machine-Generated	Machine-Generated
New Jersey	Original	Original
New Mexico	None	Machine-Generated
New York	Original	Original
North Carolina	Machine-Generated and Original	Machine-Generated and Original
North Dakota	Original	Original^b^
Ohio	Machine-Generated	Machine-Generated
Oklahoma	Machine-Generated and Original	Machine-Generated and Original
Oregon	Machine-Generated and Original	Machine-Generated and Original
Pennsylvania	Machine-Generated	Machine-Generated
Rhode Island	Original	Original
South Carolina	Machine-Generated and Original	Machine-Generated and Original
South Dakota	Original	None
Tennessee	Machine-Generated	Machine-Generated
Texas	Original	Original
Utah	Original	Original
Vermont	Machine-Generated and Original	Machine-Generated
Virginia	Machine-Generated and Original	Original
Washington	Original	Original
West Virginia	None	None
Wisconsin	Original	None
Wyoming	Machine-Generated and Original	Machine-Generated

^a^ VAMS registration only.

^b^ PrepMod registration only.

### Language coverage of COVID-19 vaccine websites

We next assessed the language coverage of each jurisdiction's multilingual COVID-19 vaccine resources. We compared the professional translations provided by jurisdictions with the languages “expected” based on three population thresholds: 5%, 0.5%, and 0.07% (see Methods section).

#### Expected languages

**FIG. 1.**
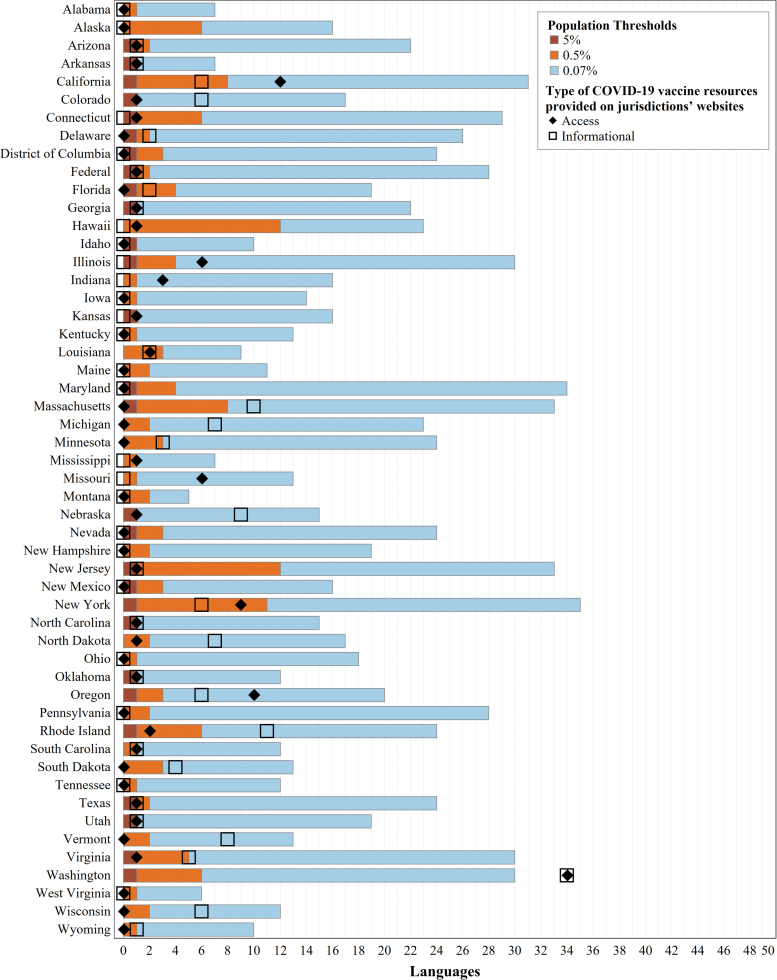
Language coverage of COVID-19 vaccine websites by jurisdiction. This chart shows the number of languages provided through professional translation of COVID-19 vaccine informational or access resources on each jurisdiction's COVID-19 website, relative to the number of non-English languages expected based on several population thresholds. The length of each colored bar is cumulative, where the total length of each bar (*brown*, *orange*, and *blue* sections) is the total number of non-English languages spoken at home by at least 0.07% of a jurisdiction's population (per language). The number of non-English languages spoken at home by at least 0.5% of a jurisdiction's population is indicated by the total length of the *brown* and *orange* bars, and the total number of non-English languages spoken at home by at least 5% of a jurisdiction's population is shown by the length of the *brown* bar. For each jurisdiction, the number of languages provided for vaccine informational resources is designated by an *open square*, and the number of languages provided for vaccine access resources is designated by a *diamond*. COVID-19, coronavirus disease 2019.

Using the 5% threshold, 24 jurisdictions do not require any translation and the remaining 28 jurisdictions only require Spanish. At the intermediate 0.5% threshold, an average (mean) of three languages are expected, with the greatest number of languages expected in Hawaii (13) and New Jersey (12). At the 0.07% threshold, an average of 19 languages are expected, with New York and Maryland expected to provide 35 languages each (Fig. 1).

#### Number of languages provided

We compared the number of languages for which professional translations of COVID-19 vaccine resources were provided on jurisdictions' websites with the expected number of translations for the jurisdiction (Fig. 1). Forty-four jurisdictions met their 5% threshold for informational resources, including 29 that provided Spanish translations and 15 that met this threshold because no translations were required. Twenty met their 0.5% threshold, and only Washington state met the 0.07% threshold. Regarding vaccine access resources, 44 jurisdictions met their 5% threshold (including 17 that neither provided nor required translations), 15 met their 0.5% threshold, and only Washington met the 0.07% threshold. Washington provided original translations of COVID-19 resources in the highest number of languages (34), followed by California (access resources in 12 languages) and Rhode Island (informational resources in 11 languages).

#### Specific languages provided

**FIG. 2.**
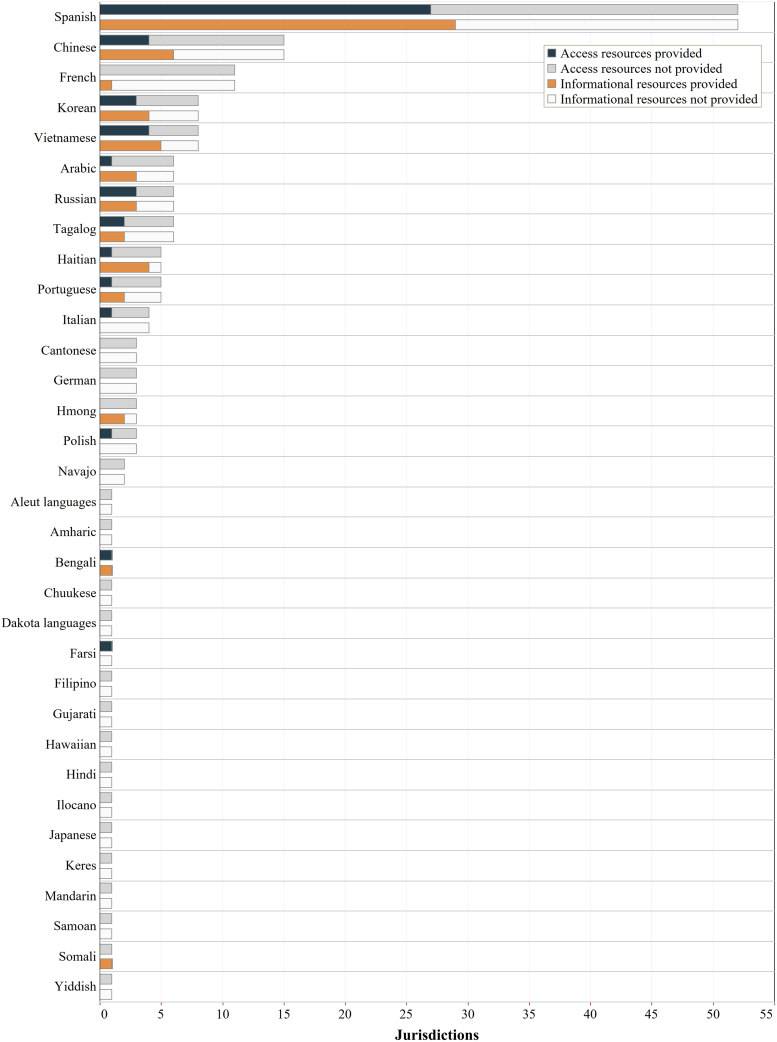
Language coverage of COVID-19 vaccine websites by language. This chart shows number of professional translations of COVID-19 vaccine informational and access resources on state/D.C./federal COVID-19 websites by language. The full bar (*black* outline) represents the total number of jurisdictions that are expected to provide a translation in that language based on a 0.5% population threshold. The colored portion of the bar represents the number of jurisdictions that provided professional translations of vaccine access resources (*navy blue*) and vaccine informational resources (*orange*) in that language, across all jurisdictions (states, D.C., and federal). The remaining portion of the bar represents the number of jurisdictions that were expected to provide professional translation in that language but did not, for access resources (*gray*) and informational resources (*white*). This chart shows all languages that are expected in one or more states/D.C. using the 0.5% population threshold, listed in descending order based on the number of jurisdictions expected to provide the language. The chart does not display languages that are spoken at home by fewer than 0.5% of the population of any individual state or D.C. D.C., District of Columbia.

We then analyzed which languages were provided through professional translations, compared with expected languages at the 0.5% population threshold (Fig. 2). Overall, 33 languages were expected in one or more jurisdictions at the 0.5% threshold. Of these, multilingual informational or access resources were provided for 16 languages in at least one jurisdiction that expected it, and 17 languages were not provided at all. Of the 16 languages provided, only 3 languages (Bengali, Farsi, and Somali) were provided for access and/or informational resources in all expected jurisdictions (1 each). The remaining 13 languages were missing in some or most jurisdictions, including the most commonly expected languages of Spanish and Chinese. At the federal level, Spanish and Chinese would be expected at the 0.5% threshold, but only Spanish was provided on the federal webpage.

### Population coverage of COVID-19 vaccine websites

**FIG. 3.**
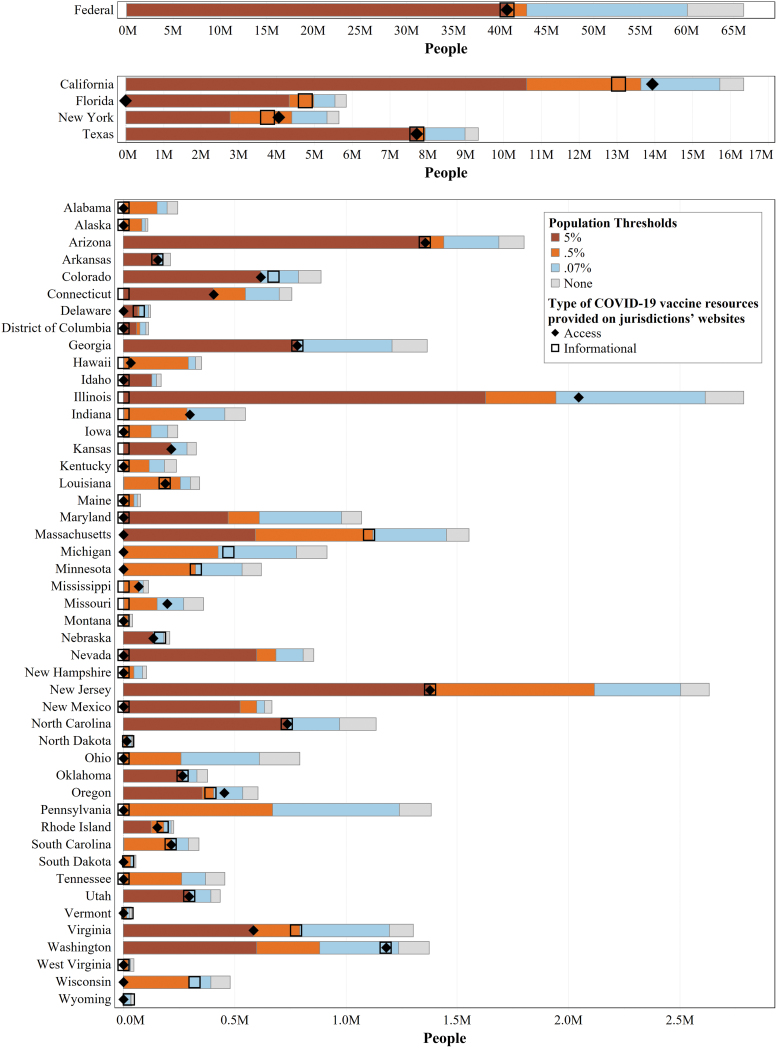
Person coverage of COVID-19 vaccine resource translations by jurisdiction. This chart shows the total number of non-English speakers in each jurisdiction, compared with the number of non-English speakers with professionally translated versions of COVID-19 vaccine resources available in their primary language on their state/D.C./federal COVID-19 website. The length of each colored portion is cumulative, where total length of the bar (*brown*, *orange*, *blue*, and *gray* sections) is the total number of people who speak a language other than English at home in the jurisdiction. The *brown* bar represents the number of people who speak a language that is spoken at home by at least 5% of the jurisdiction's population; the combined length of the *brown* and *orange* bars represents the number of people who speak a language that is spoken at home by at least 0.5% of the jurisdiction's population; and the combined length of the *brown*, *orange*, and *blue* bars represents the number of people who speak a language that is spoken at home by at least 0.07% of the jurisdiction's population. The number of people with professionally translated versions of vaccine informational resources available in their primary language from their jurisdiction's website is designated by an *open square*, and the number of people with professionally translated versions of access resources in their primary language is designated by a diamond. Data for California, Florida, New York, and Texas are shown using a separate x-axis scale than the other states/D.C. because of their much larger populations, and the federal data are shown on a separate x-axis scale from individual states/D.C. for the same reason.

We analyzed the number of people who speak a non-English language at home for whom professionally translated COVID-19 vaccine resources are available on their jurisdiction's COVID-19 website (Fig. 3). Approximately 33,000 to 16 million people per jurisdiction speak a language other than English at home, with a total of 66 million across all states and D.C. Excluding the federal website, just 40 million people had professional translations of *informational* resources available in their primary language and just 37 million people had professional translations of *access* resources available in their primary language.

We then calculated how many people would have resources available if jurisdictions were to apply population thresholds for determining translation needs. Applying the 5% threshold to the states and D.C., which only requires Spanish translations in some states, would result in 37 million Spanish speakers having COVID-19 vaccine resources available in Spanish, leaving 29 million people who speak a non-English language at home without resources in their primary language.

Applying the intermediate threshold of 0.5%, 50 million Americans would have COVID-19 vaccine resources available in their primary language—10 million more than had informational resources available and 13 million more than had access resources available, as of data capture. At the 0.07% threshold, translated resources would be available to 62 of the 66 million Americans whose primary language is not English.

On a federal level, the provided Spanish translations covered the 41 million Spanish speakers in the United States but left out 25 million individuals who speak another non-English language at home. Meeting the 0.5% and 0.07% thresholds at the federal level would make COVID-19 resources available to 43 and 60 million people, respectively.

## Discussion

Public health entities play a critical role in promoting COVID-19 vaccine uptake, as an official source of information about vaccine safety, efficacy, and access. Although state PHD websites are one of many components of COVID-19 vaccination campaigns, these websites serve an important role as a centralized, legitimate resource for combating disinformation and helping people understand how to obtain the vaccine. Consequently, providing linguistically and culturally appropriate COVID-19 vaccine information on states' COVID-19 websites, through professional translations, is an important facet of facilitating equitable access to the vaccine for non-English-communicating populations.

Our analysis showed that professional translations of PHDs' COVID-19 vaccine resources were only provided, for at least one language, in 29 jurisdictions for informational resources and 27 jurisdictions for access resources. Of those jurisdictions, most provided one or a few languages only, with commonly spoken languages such as Chinese languages, Korean, or Vietnamese often missing. As a result, 26 million and 29 million Americans who speak a non-English language at home do not have professional translations of vaccine informational and access resources, respectively, available in their primary language from their jurisdiction's COVID-19 website.

Because there is no federal standard for providing translated resources, we evaluated how various legislatively informed population thresholds would influence the number of languages states are expected to provide and the number of nonprimary English speakers helped by those languages. This analysis demonstrated the need to define these thresholds carefully, given that the most common threshold used in local and state policies (5%) would not necessitate *any* translations in 24 jurisdictions. However, the most stringent threshold (0.07%) may be unrealistic to achieve in many jurisdictions, as it would require up to 35 languages in some jurisdictions. Depending on population size, language diversity, and resources available to PHDs, an intermediate threshold, expressed as a population percentage or numerical threshold, may be more appropriate and feasible.

States providing professionally translated COVID-19 vaccine resources in the greatest number of languages have varying language access policies. Washington state (34 languages) released a Language Access Plan specifically for the COVID-19 response, requiring state agencies to translate “vital information” for each language group that constitutes at least 5% of the agency's target population or 1000 people (whichever is less).^14,33^ The Plan describes a process for centralized coordination of translation by a state agency to ensure accuracy, consistency, and timeliness and also commits state resources for the provision of translations. California (12 languages) and Massachusetts (10 languages), both have general state language access laws that apply to all state agencies and use a 5% population threshold, whereas Rhode Island (11 languages) does not have a state-specific language access policy.^10,13^

Further evaluation of states' language access policies and plans, in light of their successes and challenges for translation of COVID-19 resources, could yield insights into the types of policies and practices that best enable states to provide timely and accurate translations of critical health information.

Our study did not assess the quality or accessibility of translated information about COVID-19 vaccines. Although some translated resources were equivalent to the English-language versions, many provided less and/or older information. Furthermore, on some PHD websites, translated resources were difficult to find. Other studies have also documented reduced content, readability, and ease of navigability of the non-English parts of the COVID-19 websites of selected PHDs and private health care systems.^16,34^ These findings highlight the need for additional studies on the accessibility of translated COVID-19 information, which could inform the development of translation standards and guide the design of more user-friendly websites for non-English speakers.

Our study has several limitations. First, the study only analyzes online, text-based informational and access resources for COVID-19 vaccines on states' COVID-19 websites. Many states also provide informational videos in non-English languages, offer interpretation through phone, and/or rely on local PHDs to provide online resources.

Second, although we investigated multiple facets of each website to assess whether translations were professional or machine generated, we were unable to confirm our assessments with all state PHDs. As a result, we may have overestimated the number of professional translations by counting an available translation as professional when it may have been machine generated.

Finally, our analysis is based on ACS data on individuals who speak a language other than English at home, rather than individuals with LEP, because the only per-language LEP data were outdated (2009–2013). Consequently, the number of people who may *require* access to COVID-19 vaccine websites in a non-English language is likely lower than estimated in our analysis. This limitation can likely be mitigated with upcoming ACS releases of data on Americans with LEP.

Our findings highlight the importance of allocating sufficient resources to PHDs for providing professional translations of COVID-19 vaccine resources on their websites to reach vulnerable populations. Although the use of free machine-generated translation services is appealing given competing time and budget demands during an emergency response, machine translations can be technically inaccurate and culturally insensitive.

To ensure availability of accurate, up-to-date vaccine information, PHDs need resources to translate key information in multiple languages, integrate translated content into their websites, and update this information regularly to adequately serve their populations. Our analysis provides estimates and a methodology that PHDs can use to determine and justify with data the number of languages for which they should provide translations, which can be used to calculate resource needs and plan for how to provide translations in a timely manner. Analysis of common language needs across states also could inform efforts to create common resources that could be disseminated by multiple states, coordinated at the federal and/or regional level.

## Conclusion

This analysis of the availability of professionally translated vaccine information on states' COVID-19 websites informs discussions about resource needs and operational considerations for making critical health information available to non-English-communicating Americans in their primary languages. To holistically understand the effectiveness of and needs for online dissemination of COVID-19 vaccine information to this population, further study is needed to assess the quality and extent of translations provided by local PHDs, third-party vaccine providers, and community organizations, as well as to evaluate the accessibility of online information provided by states. In the near term, reevaluating what language resources are needed will be useful for the remainder of the COVID-19 vaccination campaigns, as many U.S. adults and children are not yet vaccinated or boosted. In the longer term, language translation needs should be incorporated into planning for future pandemics or public health crises, so that governments can respond nimbly to disseminate crucial resources to non-English-communicating populations when emergencies arise.

## Supplementary Material

Supplemental data

## Abbreviations Used

ACS: American Community Survey
COVID-19: coronavirus disease 2019
D.C.: District of Columbia
GT: Google Translate
LEP: limited English proficiency
PHD: public health department
