# Supplementary material for: Analysis of Language Translations of State Governments' Coronavirus Disease 2019 Vaccine Websites
Source: Health Equity. 2022 Sep 22;6(1):738–49. doi: 10.1089/heq.2021.0189 (PMC9536349; doi:10.1089/heq.2021.0189)
Supplement: Supplemental data [file Supp_TableS1.pdf]

	Informational Webpages	Access Webpages	Registration	Exclusion notes
**Alabama**	https://web.archive.org/web/20211012114636/https://www.alabamapublichealth.gov/covid19vaccine/basics.html	https://web.archive.org/web/20211010133600/https://www.alabamapublichealth.gov/covid19vaccine/index.html; https://web.archive.org/web/20210918032046/https://dph1.adph.state.al.us/covid-19/	None	One sentence in Spanish referring to the WhatsApp was deemed insufficient because it was significantly less than what was provided in English
**Alaska**	https://web.archive.org/web/20211012115228/https://dhss.alaska.gov/dph/Epi/id/Pages/COVID-19/VaccineInfo.aspx	https://web.archive.org/web/20211012115302/https://dhss.alaska.gov/dph/Epi/id/Pages/COVID-19/vaccineappointments.aspx	https://web.archive.org/web/20211012115311/https://myhealth.alaska.gov/appointment/en/clinic/search	We excluded the registration site because it was no longer possible to link to it from the main informational or access pages
**Arizona**	https://web.archive.org/web/20211012115531/https://www.azdhs.gov/covid19/es/index.php	https://web.archive.org/web/20211012115546/https://www.azdhs.gov/covid19/es/vaccines/index.php	https://web.archive.org/web/20210911044902/https://podvaccine.azdhs.gov/	NA
**Arkansas**	https://web.archive.org/web/20211012120038/https://www.healthy.arkansas.gov/programs-services/topics/covid-19-for-spanish-speaking-arkansans	https://web.archive.org/web/20211012120038/https://www.healthy.arkansas.gov/programs-services/topics/covid-19-for-spanish-speaking-arkansans; https://web.archive.org/web/20211012120130/https://www.healthy.arkansas.gov/programs-services/topics/adh-covid-19-vaccine-clinics	None	NA
**California**	https://web.archive.org/web/20211012120433/https://covid19.ca.gov/es/vaccines/	https://web.archive.org/web/20211012120316/https://myturn.ca.gov/en_US.html	https://web.archive.org/web/20211012120316/https://myturn.ca.gov/en_US.html	NA
**Colorado**	https://web.archive.org/web/20211012120629/https://covid19.colorado.gov/Learn-about-COVID-19-vaccines; https://web.archive.org/web/20211012120610/https://covid19.colorado.gov/vaccine-faq; https://web.archive.org/web/20211011032701/https://covid19.colorado.gov/espanol/preguntas-frecuentes-sobre-la-vacuna	https://web.archive.org/web/20211012120709/https://covid19.colorado.gov/vaccine/where-you-can-get-vaccinated; https://covid19.colorado.gov/espanol/informese-donde-puede-vacunarse	None	NA

**Connecticut**	https://web.archive.org/web/20211012121028/https://portal.ct.gov/vaccine-portal/Vaccine-Knowledge-Base/Categories/COVID-19-Vaccine?language=en_US	https://web.archive.org/web/20211012121313/https://portal.ct.gov/Vaccine-Portal?language=en_US; https://web.archive.org/web/20211007153458/https://covidvaccinefinder.ct.gov/	https://web.archive.org/web/20211012121351/https://guest.vams.cdc.gov/guest?lang=en	NA
**Delaware**	https://web.archive.org/web/20211012122028/https://coronavirus.delaware.gov/vaccine/about-the-science/; https://web.archive.org/web/20211012122043/https://coronavirus.delaware.gov/frequently-asked-questions/		None	Resources were provided in Spanish and Hatian Creole. These were deemed sufficient for information, but insufficient for access, due to the fact that they did not provide the same links as in English or the information about walk-up vaccine sites
**District of Columbia**	https://web.archive.org/web/20211012122932/https://coronavirus.dc.gov/vaccine	https://web.archive.org/web/20211012122958/https://coronavirus.dc.gov/page/get-vaccinated	None	NA
**Federal**	https://web.archive.org/web/20211012123107/https://www.vaccines.gov/	https://web.archive.org/web/20211012123125/https://www.vaccines.gov/search/	None	NA
**Florida**	https://web.archive.org/web/20211012123431/https://floridahealthcovid19.gov/vaccines/	https://web.archive.org/web/20211012123458/https://floridahealthcovid19.gov/vaccines/vaccine-locator/	None	NA
**Georgia**	https://web.archive.org/web/20211012123734/https://dph.georgia.gov/covid-19-vaccine-general-faq	https://web.archive.org/web/20211012124453/https://dph.georgia.gov/covid-vaccine	https://web.archive.org/web/20210927053743/https://gta-vras.powerappsportals.us/en-US/	NA
**Hawaii**	https://web.archive.org/web/20211012125146/https://hawaiicovid19.com/vaccine-faqs/	https://web.archive.org/web/20211012125159/https://hawaiicovid19.com/vaccine/	https://web.archive.org/web/20211012121351/https://guest.vams.cdc.gov/guest?lang=en	NA
**Idaho**	https://web.archive.org/web/20210912175251/https://healthandwelfare.idaho.gov/idaho-covid-19-vaccination-information/covid-19-vaccine-faq; https://web.archive.org/web/20211012132145/https://coronavirus.idaho.gov/covid-19-vaccine/	https://web.archive.org/web/20211012132152/https://healthandwelfare.idaho.gov/covid-19-vaccination; https://web.archive.org/web/20211012132203/https://idaho.maps.arcgis.com/apps/dashboards/37170663dc9441e2b6d7c1dba6135357	None	The only Spanish resource found was a map for mobile COVID-19 vaccination clinics; however, the authors were never able to get the website to load properly.

**Illinois**	https://web.archive.org/web/20211012132457/https://dph.illinois.gov/covid19/vaccine-faq	https://web.archive.org/web/20211008224517/https://coronavirus.illinois.gov/vaccines/vaccination-locations.html; https://web.archive.org/web/20210927013750/https://coronavirus.illinois.gov/content/dam/soi/en/web/coronavirus/documents/vax-locations-spanish.pdf; https://web.archive.org/web/20211012132948/https://coronavirus.illinois.gov/content/dam/soi/en/web/coronavirus/documents/vax-locations-chinese.pdf	https://web.archive.org/web/20211012133123/https://covidvaccination.dph.illinois.gov/	NA
**Indiana**	https://web.archive.org/web/20211012134751/https://www.coronavirus.in.gov/vaccine/	https://web.archive.org/web/20211011023240/https://experience.arcgis.com/experience/24159814f1dd4f69b6c22e7e87bca65b	https://web.archive.org/web/20211012135017/https://vaccine.coronavirus.in.gov/en-US/	There was a note above the map in Spanish that you could call for assistance, but this was deemed insufficient.
**Iowa**	https://web.archive.org/web/20211012135356/https://vaccinate.iowa.gov/know-the-facts/; https://web.archive.org/web/20211012135339/https://vaccinate.iowa.gov/about-covid-19-vaccine/	https://web.archive.org/web/20211012135313/https://vaccinate.iowa.gov/providers/	None	NA
**Kansas**	https://web.archive.org/web/20211006094654/https://www.kansasvaccine.gov/189/Habitantes-de-Kansas; https://web.archive.org/web/20211012141828/https://www.coronavirus.kdheks.gov/284/COVID-19-Vaccine	https://web.archive.org/web/20211006091042/https://www.kansasvaccine.gov/191/Encuentra-Mi-Vacuna	None	The informational webpage in Spanish was excluded because it provided very little information about the vaccine, especially in comparison to the English website, and it linked to outside English resources. We note that it does link to some informational videos in multiple languages.
**Kentucky**	https://web.archive.org/web/20211011080635/https://govstatus.egov.com/ky-covid-vaccine	https://web.archive.org/web/20211011080635/https://govstatus.egov.com/ky-covid-vaccine	None	NA

**Louisiana**	https://web.archive.org/web/20211011080637/https://ldh.la.gov/covidvaccine/; https://web.archive.org/web/20211012142240/https://ldh.la.gov/assets/oph/Coronavirus/marketingmaterials/vt/COVID_Vaccine_FAQs_VI.pdf; https://web.archive.org/web/20210916161930/https://ldh.la.gov/assets/oph/Coronavirus/marketingmaterials/sp/COVID_Vaccine_FAQs_ES.pdf	https://web.archive.org/web/20211006142858/https://ldh.la.gov/directory/vaccination; https://web.archive.org/web/20211012142240/https://ldh.la.gov/assets/oph/Coronavirus/marketingmaterials/vt/COVID_Vaccine_FAQs_VI.pdf; https://web.archive.org/web/20210916161930/https://ldh.la.gov/assets/oph/Coronavirus/marketingmaterials/sp/COVID_Vaccine_FAQs_ES.pdf	None	NA
**Maine**	https://web.archive.org/web/20211010153049/https://www.maine.gov/covid19/vaccines; https://web.archive.org/web/20211011080635/https://www.maine.gov/covid19/vaccines/public-faq	https://web.archive.org/save/https://www.maine.gov/covid19/vaccines/vaccination-sites	https://web.archive.org/web/20210924151757/https://vaccinateme.maine.gov/	We excluded the registration site because it was no longer possible to link to it from the main informational or access pages
**Maryland**	https://web.archive.org/web/20211012142855/https://covidlink.maryland.gov/content/vaccine/; https://web.archive.org/web/20210916124026/https://covidlink.maryland.gov/content/faqs/	https://web.archive.org/web/20211010135936/https://coronavirus.maryland.gov/pages/vaccine	None	NA
**Massachusetts**	https://web.archive.org/web/20211012143449/https://www.mass.gov/info-details/trust-the-facts-get-the-vax; https://web.archive.org/web/20211012143453/https://www.mass.gov/info-details/covid-19-vaccine-frequently-asked-questions; https://web.archive.org/web/20211012143457/https://www.mass.gov/doc/vacuna-contra-el-covid-19-preguntas-frecuentes/download	http://web.archive.org/web/20211010044251/https://vaxfinder.mass.gov/; http://web.archive.org/web/20211010161201/https://www.mass.gov/info-details/covid-19-vaccination-locations	None	NA

**Michigan**	https://web.archive.org/web/20211012144132/https://www.michigan.gov/coronavirus/0,9753,7-406-98178_98541_98542---,00.html; https://web.archive.org/web/20210627052711/https://www.michigan.gov/documents/coronavirus/COVID19_Vaccine_FAQ_Spanish_Dec_2020_712325_7.pdf; https://web.archive.org/web/20211012144302/https://www.michigan.gov/coronavirus/0,9753,7-406-98178_98541---,00.html	https://web.archive.org/web/20211011091720/https://www.michigan.gov/coronavirus/0,9753,7-406-98178_103214---,00.html	None	PDF resources were provided in multiple languages; however, the type of resources varied per language. Languages were included only if they provided information about the safety and efficacy of the vaccine.

Minnesota	https://web.archive.org/web/20211018112929/https://www.health.state.mn.us/diseases/coronavirus/materials/spanish.html	https://web.archive.org/web/20211018113258/http://validate.perfdrive.com/d5bd5333eafe8b0ccd6023ba818d1aa6/?ssa=acc0fd78-9ed3-47b9-9b17-b410cde15545&ssb=18378222281&ssc=https%3A%2F%2Fmn.gov%2Fcovid19%2Fvaccine%2Ffind-vaccine%2Flocations%2Findex.jsp&ssi=dfb55c0f-bf56-4923-821c-26c40eedbacf&ssk=support@shieldsquare.com&ssm=63734660753544162107469992282026&ssn=12aaf0f06376fb4a3468347418cdfc3a953eca6591ca-d45c-4e3e-b87463&sso=14d89323-2f1a10b3bbfb5ae7450e98bf23011c8f3fb216a95831bb54&ssp=75181887191634590073163454291509484&ssq=39138805677793484481156777422706788991323&ssr=MjA3LjI0MS4yMjUuMjQ2&sst=Mozilla/5.0%20(Windows%20NT%2010.0;%20Win64;%20x64)%20AppleWebKit/537.36%20(KHTML,%20like%20Gecko)%20Chrome/74.0.3729.169%20Safari/537.36&ssv=&ssw=; https://web.archive.org/web/20211018113309/https://vaccineconnector.mn.gov/es-ES/; https://web.archive.org/web/20211015093536/https://www.health.state.mn.us/diseases/coronavirus/vaccine/2dosepfizerspa.pdf	https://web.archive.org/web/20211013113641/https://prepmod.health.state.mn.us/	While they provided a lot of informational resources, the access resources were lacking. The provided PDF had limited information on how to get the second dose only, and the vaccineconnector website transitioned back to English when searching.
Mississippi	https://web.archive.org/web/20211013123720/https://msdh.ms.gov/msdhsite/_static/14,22816,420,976.html	https://web.archive.org/web/20211013123729/https://msdh.ms.gov/msdhsite/_static/14,0,420,976.html	https://web.archive.org/web/20211013123832/https://covidvaccine.umc.edu/	registration are translated into Spanish, questions regarding vaccine eligibility were in English; therefore, the registration translation was excluded.
Missouri	https://web.archive.org/web/20211018114434/https://covidvaccine.mo.gov/facts/	https://web.archive.org/web/20211018114449/https://covidvaccine.mo.gov/find/	None	NA

**Montana**	https://web.archive.org/web/20211018114631/https://dphhs.mt.gov/covid19vaccine/index	https://web.archive.org/web/20211018114631/https://dphhs.mt.gov/covid19vaccine/index	None	NA
**Nebraska**	https://web.archive.org/web/20211018115216/https://dhhs.ne.gov/Pages/COVID-19-Vaccine-Information.aspx; https://web.archive.org/web/20211018115238/https://dhhs.ne.gov/Documents/COVID-19-Vaccine-FAQ-Spanish.pdf	https://web.archive.org/web/20211018115236/https://dhhs.ne.gov/Pages/FinishStrong.aspx; https://web.archive.org/web/20211018120645/https://dhhs.ne.gov/Pages/COVID-19-Information-in-Other-Languages.aspx	https://web.archive.org/web/20210331005028/https://vaccinate.ne.gov/es-ES/	NA
**Nevada**	https://web.archive.org/web/20211018115826/https://www.nvcovidfighter.org/covid-19-faq	https://web.archive.org/web/20211018115740/https://www.nvcovidfighter.org/covid-19-vaccine-locator; https://web.archive.org/web/20211011175730/https://www.nvcovidfighter.org/county-specific-covid-19-vaccine-sites	None	NA
**New Hampshire**	https://web.archive.org/web/20210720220443/https://www.vaccines.nh.gov/?vaccinated	https://web.archive.org/web/20211018121043/https://www.vaccines.nh.gov/helpful-info/recipient-questions	https://web.archive.org/web/20211018121010/https://vini.nh.gov/providers/s/	it was no longer possible to link to it from the main informational or access pages
**New Jersey**	https://web.archive.org/web/20211018121728/https://covid19.nj.gov/pages/vaccine; https://web.archive.org/web/20211018121735/https://covid19.nj.gov/es/pages/vaccine	https://web.archive.org/web/20211018121728/https://covid19.nj.gov/pages/vaccine; https://web.archive.org/web/20211018121735/https://covid19.nj.gov/es/pages/vaccine	https://web.archive.org/web/20211018122035/https://covidvaccine.nj.gov/en-US/	NA
**New Mexico**	https://web.archive.org/web/20211018123557/https://cv.nmhealth.org/covid-vaccine/	https://web.archive.org/web/20211015235709/https://goodtimes.vaccinenm.org/stay-ahead-nm/; https://web.archive.org/web/20210914130423/https://cv.nmhealth.org/wp-content/uploads/2021/03/2021.1.28-DOH-Phase-Guidance1SPOz-1.pdf	https://web.archive.org/web/20211018123641/https://vaccinenm.org/my-registration.html	NA

**New York**	https://web.archive.org/web/20211013072219/https://covid19vaccine.health.ny.gov/frequently-asked-questions-0; https://web.archive.org/web/20210909000334/https://covid19vaccine.health.ny.gov/system/files/documents/2021/02/covid-19vaccine_faq_spanish.pdf	https://web.archive.org/web/20211013072219/https://covid19vaccine.health.ny.gov/frequently-asked-questions-0; https://web.archive.org/web/20210909000334/https://covid19vaccine.health.ny.gov/system/files/documents/2021/02/covid-19vaccine_faq_spanish.pdf	https://web.archive.org/web/20211014193705/https://am-i-eligible.covid19vaccine.health.ny.gov/Public/prescreener	NA
**North Carolina**	https://web.archive.org/web/20211015133306/https://covid19.ncdhhs.gov/vaccines/frequently-asked-questions-about-covid-19-vaccinations; https://web.archive.org/web/20211018124139/https://covid19.ncdhhs.gov/vaccines/en-espanol/preguntas-frecuentes	https://web.archive.org/web/20211015133504/https://covid19.ncdhhs.gov/vaccines/informacion-sobre-las-vacunas-contra-el-covid-19; https://web.archive.org/web/20211018034242/https://covid19.ncdhhs.gov/vaccines	None	NA
**North Dakota**	https://web.archive.org/web/20211018125053/https://www.health.nd.gov/nfi-covid-translations	https://web.archive.org/web/20211018125057/https://www.health.nd.gov/covid-vaccine-locator	https://web.archive.org/web/20211018124709/https://www.ndvax.org/	NA
**Ohio**	https://web.archive.org/web/20211018130252/https://coronavirus.ohio.gov/wps/portal/gov/covid-19/covid-19-vaccination-program/covid-19-vaccine-myths-vs-facts/covid-19-vaccine-myths-vs-facts; https://web.archive.org/web/20211018130134/https://coronavirus.ohio.gov/static/vaccine/covid-19-vaccine-myth-vs-facts-sp.pdf	https://web.archive.org/web/20211018125713/https://coronavirus.ohio.gov/wps/portal/gov/covid-19/covid-19-vaccination-program/covid-19-vaccination-program/covid-19-vaccination-program	https://web.archive.org/web/20211017171508/https://gettheshot.coronavirus.ohio.gov/	NA

**Oklahoma**	https://web.archive.org/web/20211018132047/https://oklahoma.gov/covid19/vaccine-information/vaccine-faqs.html; https://web.archive.org/web/20211017033135/https://oklahoma.gov/covid19/vaccine-information/about-the-vaccine.html; https://web.archive.org/web/20210914145404/https://oklahoma.gov/content/dam/ok/en/covid19/documents/vaccine/Spanish%20COVID-19%20Vaccine%20FAQs.pdf	https://web.archive.org/web/20211018034240/https://oklahoma.gov/covid19/vaccine-information.html	https://web.archive.org/web/20211018132103/https://vaccinate.oklahoma.gov/en-US/	NA
**Oregon**	https://web.archive.org/web/20211018034242/https://covidvaccine.oregon.gov/; https://web.archive.org/web/20211016234423/https://govstatus.egov.com/or-oha-vaccine-faqs	https://web.archive.org/web/20211016233729/https://govstatus.egov.com/find-covid-19-vaccine; https://web.archive.org/web/20211016234423/https://govstatus.egov.com/or-oha-vaccine-faqs	None	Languages that use non-roman alphabet are not rendering properly on any browser and are therefore excluded.
**Pennsylvania**	https://web.archive.org/web/20211012170951/https://www.health.pa.gov/topics/disease/coronavirus/Vaccine/Pages/FAQs.aspx	https://web.archive.org/web/20211018134001/https://www.pa.gov/guides/get-vaccinated/; https://web.archive.org/web/20211018034245/https://www.health.pa.gov/topics/disease/coronavirus/Vaccine/Pages/Vaccine.aspx	None	NA
**Rhode Island**	https://web.archive.org/web/20211018143327/https://covid.ri.gov/vaccination/covid-19-vaccine-faqs?language=en; https://web.archive.org/web/20211018143327/https://covid.ri.gov/vaccination/covid-19-vaccine-faqs?language=en; https://web.archive.org/web/20210910014605/https://covid.ri.gov/multiple-languages/french-francais?language=pt-pt	https://web.archive.org/web/20210910153713/https://covid.ri.gov/vaccination?language=en; https://web.archive.org/web/20211018143555/https://covid.ri.gov/vaccination?language=es	https://web.archive.org/web/20211018143604/https://www.vaccinateri.org/	NA

**South Carolina**	https://web.archive.org/web/20211018143849/https://scdhec.gov/covid19/covid-19-vaccine/covid-19-vaccine-faqs; https://web.archive.org/web/20211018143854/https://scdhec.gov/covid19/covid-19-vaccine; https://web.archive.org/web/20211018143903/https://scdhec.gov/covid19-es/vacuna-contra-la-covid-19	https://web.archive.org/web/20211018143913/https://vaxlocator.dhec.sc.gov/?l=en; http://web.archive.org/web/20210803095859/https://vaxlocator.dhec.sc.gov/?l=es	None	NA
**South Dakota**	https://web.archive.org/web/20211015235331/https://doh.sd.gov/documents/COVID19/Vaccine/WCDT_VaccineInformation_Spanish.pdf; https://web.archive.org/web/20211017140440/https://doh.sd.gov/COVID/Vaccine/faqs.aspx; https://web.archive.org/web/20210907233755/https://covid.sd.gov/ESL.aspx	https://web.archive.org/web/20211017104529/https://doh.sd.gov/COVID/Vaccine/ProviderMap/default.aspx	None	NA
**Tennessee**	https://web.archive.org/web/20211017060819/https://covid19.tn.gov/covid-19-vaccines/vaccine-faqs/	https://web.archive.org/web/20211018145416/https://covid19.tn.gov/covid-19-vaccines/availability/	None	While some resources about COVID-19 were provided in Spanish, they did not relate to the COVID-19 vaccine.
**Texas**	https://web.archive.org/web/20211014012834/https://dshs.texas.gov/coronavirus/immunize/vaccine-faqs-sp.aspx	https://web.archive.org/web/20211018145855/https://dshs.texas.gov/covidvaccine/; https://web.archive.org/web/20210910194338/https://dshs.texas.gov/coronavirus/immunize/vaccine-sp.aspx	https://getthevaccine.dshs.texas.gov/txces/s/?language=en_US	NA
**Utah**	https://web.archive.org/web/20211016203036/https://coronavirus.utah.gov/espanol; https://web.archive.org/web/20211005022437/https://coronavirus.utah.gov/vacunas/	https://web.archive.org/web/20211018151536/https://coronavirus.utah.gov/distribucion-de-vacunas/	None	Utah appears as an option on the VAMS website, but there is no link from the public health website and when trying to make an appointment, it showed no appointments available.

**Vermont**	https://web.archive.org/web/20211017191430/https://www.healthvermont.gov/covid-19/faqs; https://web.archive.org/web/20211018153103/https://www.healthvermont.gov/covid-19/vaccine; https://web.archive.org/web/20210917060623/https://www.healthvermont.gov/sites/default/files/documents/pdf/COVID19-Vaccine-FAQ-Handout.pdf	https://web.archive.org/web/20211018152907/https://www.healthvermont.gov/covid-19/vaccine/getting-covid-19-vaccine	https://web.archive.org/web/20210922191324/https://vermont.force.com/events/s/selfregistration	NA
**Virginia**	https://web.archive.org/web/20211018154331/https://www.vdh.virginia.gov/covid-19-vaccine/; https://web.archive.org/web/20211018154336/https://www.vdh.virginia.gov/covid-19-faq/vaccination/; https://web.archive.org/web/20211009223708/https://www.vdh.virginia.gov/content/uploads/sites/198/2021/01/Spanish_Vaccination.pdf	https://web.archive.org/web/20211017040526/https://vaccinate.virginia.gov/	https://web.archive.org/web/20211012121351/https://guest.vams.cdc.gov/guest?lang=en	NA
**Washington**	https://web.archive.org/web/20211012105444/https://www.doh.wa.gov/Emergencies/COVID19/VaccineInformation/VaccineFacts; https://web.archive.org/web/20211018155231/https://www.doh.wa.gov/Emergencies/COVID19/VaccineInformation/VaccineFacts/Spanish; https://web.archive.org/web/20211013002105/https://www.doh.wa.gov/Emergencies/COVID19	https://web.archive.org/web/20211015235303/https://vaccinelocator.doh.wa.gov/	None	NA
**West Virginia**	https://web.archive.org/web/20211018155714/https://dhhr.wv.gov/COVID-19/Pages/Vaccine.aspx	https://web.archive.org/web/20211018155611/https://dhhr.wv.gov/News/2021/Pages/Upcoming-COVID-19-Vaccine-Clinics.aspx	https://web.archive.org/web/20211012121351/https://guest.vams.cdc.gov/guest?lang=en	We excluded the registration site because it was no longer possible to link to it from the main informational or access pages

**Wisconsin**	https://web.archive.org/web/20211018151945/https://www.dhs.wisconsin.gov/covid-19/index.htm; https://web.archive.org/web/20211003235500/https://www.dhs.wisconsin.gov/covid-19/resources.htm;	https://web.archive.org/web/20211019175429/https://www.dhs.wisconsin.gov/covid-19/vaccine-get.htm; https://web.archive.org/web/20211019175409/https://211wisconsin.communityos.org/public-event-search?localHistory=Z9hrfYwS-BSy-GHFTZwq4g	None	We excluded the registration site because it was no longer possible to link to it from the main informational or access pages
**Wyoming**	https://web.archive.org/web/20211019041741/https://health.wyo.gov/publichealth/immunization/wyoming-covid-19-vaccine-information/; https://web.archive.org/web/20210824165917/https://health.wyo.gov/wp-content/uploads/2021/03/VaccineDecisionsSpanish_3-22.pdf; https://web.archive.org/web/20210824165347/https://health.wyo.gov/wp-content/uploads/2021/06/datos-sobre-la-vacunacion-contra-covid19-para-padres.pdf	https://web.archive.org/web/20211010105851/https://health.wyo.gov/publichealth/immunization/wyoming-covid-19-vaccine-information/county-covid-19-vaccine-information/	None	NA
